# Evaluating the role of craniocervical and atlantoaxial instability in autonomic dysfunction: a narrative review

**DOI:** 10.3389/fnins.2026.1967117

**Published:** 2026-10-26

**Authors:** Louis Vilotti, Nicholas DePace, Joseph Colombo, Adam Benn

**Affiliations:** Franklin Cardiovascular Associates, Sicklerville, NJ, United States

**Keywords:** atlantoaxial instability, autonomic nervous, autonomic dysfunction, autonomic nervous system, cervical instability, craniocervical instability, dysautonomia, hypermobile Ehlers Danlos syndrome

## Abstract

**Background:**

Autonomic dysfunction is a complex condition that affects every organ system in the body. The underlying mechanisms of autonomic dysfunction remain incompletely understood. There appears to be an association between patients with hypermobile Ehlers Danlos syndrome (hEDS), craniocervical instability (CCI), atlantoaxial instability (AAI), and autonomic dysfunction. There is biological plausibility for CCI and AAI being causes for autonomic dysfunction in these populations.

**Objective:**

The objective of this review is to explore the literature on the potential association between CCI, AAI and autonomic dysfunction and to explore the biological plausibility of this association.

**Methods:**

A narrative review of PubMed was conducted through August 2026 to evaluate associations among hEDS, CCI/AAI, and autonomic dysfunction. Studies addressing autonomic dysfunction in hypermobility disorders, CCI/AAI, autonomic outcomes following cervical stabilization surgeries, and proposed neurovascular or autonomic mechanisms were included. Findings were synthesized narratively due to the heterogeneity of the study designs analyzed and outcome measures assessed.

**Results:**

43 studies were included in the final review. Evidence consistently supported an association between hEDS and autonomic dysfunction and between hEDS and CCI/AAI. More limited evidence demonstrated a direct overlap between CCI/AAI and dysautonomia, while multiple series reports showed improvements in autonomic-associated symptoms following surgical stabilization procedures. Proposed mechanisms for autonomic dysfunction in CCI/AAI patients include vagal nerve involvement, internal jugular vein compression, altered cerebral venous outflow, and cervicomedullary effects. The evidence was predominantly retrospective and heterogeneous, with limited standardized autonomic testing, warranting further investigation and more robust future analysis.

**Conclusion:**

Current evidence suggests that CCI and AAI represent under-recognized contributors to dysautonomia. While causality has not been established, anatomical, physiological, and clinical observations warrant further investigation into this relationship. Standardized diagnostic criteria for CCI/AAI, as well as autonomic dysfunction, must be established to further study these relationships. Prospective studies incorporating these diagnostic criteria and objective autonomic measurements will assist in clarifying these relationships and determine what interventions will help improve patient outcomes.

## Introduction

The autonomic nervous system (ANS) regulates involuntary physiologic processes in every organ system of the body (Gibbons, 2019). Autonomic dysfunction can therefore produce multisystem manifestations, contributing to heterogeneous patient presentations (Song et al., 2020). Common presentations include orthostatic intolerance, cardiovascular symptoms, gastrointestinal dysmotility, thermoregulatory abnormalities, fatigue, and cognitive dysfunction (Peltier, 2024). Despite increasing recognition surrounding the ANS and its role in various pathological processes, much is misunderstood about how much the ANS contributes to pathology.

Consistent associations between hypermobile Ehlers-Danlos syndrome (hEDS) and autonomic dysfunction have been reported (Hakim et al., 2017a) (Novak et al., 2025; Miller et al., 2020; Celletti et al., 2020; Peebles et al., 2022). Postural orthostatic tachycardia syndrome (POTS) is commonly described in these populations, although autonomic manifestations extend beyond POTS and orthostatic intolerance (OI) (Celletti et al., 2020; Peebles et al., 2022). These manifestations may involve multiple organ systems and more diverse symptomatology (Hakim et al., 2017b; Aziz et al., 2025; Hertel et al., 2024). Many mechanisms have been hypothesized for these patients, including cardiovascular deconditioning, venous pooling, autoimmunity, and small fiber neuropathy explain some, but not all the various symptoms and presentations (Hakim et al., 2017b; Kersebaum et al., 2025; Ruiz Maya et al., 2021). The pathophysiology behind the association between autonomic dysfunction and hEDS remains incompletely defined (Hakim et al., 2017b; Kersebaum et al., 2025; Blitshteyn and Miller, 2026; Wang et al., 2021).

Hypermobile Ehlers-Danlos Syndrome is characterized by generalized joint hypermobility and varying degrees of connective tissue and ligamentous laxity. This is hypothesized to be due to improper formation of collagen and other connective tissues (Hakim, 2004). This predisposes hEDS patients to improper joint stability which commonly causes chronic joint and musculoskeletal pain (Hakim, 2004).

Craniocervical instability (CCI) and atlantoaxial instability (AAI) have been described in association with hereditary connective tissue disorders, including hEDS (Mao et al., 2022; Lohkamp et al., 2022). These conditions are often accompanied by abnormal motion at the craniocervical junction and C1–C2 articulation, respectively (Mao et al., 2022; Lohkamp et al., 2022). In patients with hEDS, ligamentous laxity may contribute to inadequate stabilization of the upper cervical spine and the development of pathological instability (Mao et al., 2022; Lohkamp et al., 2022; Hauser et al., 2026).

Given the close anatomical relationship between the upper cervical spine and surrounding nerves, arteries and veins, it is plausible that chronic instability in these joints can lead to the compression and distortion of surrounding neurovascular structures (Henderson Sr et al., 2019). Excessive or abnormal motion may affect the cervicomedullary junction, vagus nerve, internal jugular veins, cerebral venous outflow, or cerebral perfusion (Midtlien et al., 2024; Henderson Sr et al., 2019; Hauser et al., 2025a). These mechanisms provide a hypothetical explanation for autonomic manifestations in this subset of patients with hESD. However, the clinical significance of these relationships remains uncertain and understudied.

This narrative review is intended to examine the available evidence linking hEDS, CCI/AAI, and autonomic dysfunction, while paying particular attention to clinical associations and outcomes following stabilization. We also aim to identify limitations that exist in the current literature and propose standardization of diagnostic criteria to further investigate these relationships.

## Methods

### Literature search strategy

A narrative review of the literature was conducted to explore the potential relationship between hypermobile Ehlers-Danlos syndrome (hEDS), hypermobility spectrum disorder (HSD), craniocervical instability (CCI), atlantoaxial instability (AAI), and autonomic dysfunction, with an emphasis on proposed neurovascular compression.

PubMed was searched through August 2026. No formal risk-of-bias or quality assessment was performed due to the nature of this being a narrative review. Only English-language studies were included for this review. The PubMed search strategy used combinations of the following keywords: “craniocervical instability,” “atlantoaxial instability,” “cervical instability,” “hypermobile Ehlers-Danlos syndrome,” “hEDS,” “autonomic dysfunction,” and “dysautonomia.” Individual terms and combinations of terms were searched to capture literature examining the relationships among cervical instability, connective tissue hypermobility, and autonomic dysfunction. Search combinations included “craniocervical instability AND dysautonomia,” “craniocervical instability AND autonomic dysfunction,” “craniocervical instability AND Ehlers-Danlos syndrome,” “atlantoaxial instability AND dysautonomia,” “atlantoaxial instability AND autonomic dysfunction,” “cervical instability AND dysautonomia,” “cervical instability AND autonomic dysfunction,” “cervical instability AND hEDS,” and “hypermobile Ehlers-Danlos syndrome AND autonomic dysfunction.”

Following the initial database search, records were screened for relevance based on the title of the manuscript and the abstract. Articles that were selected as being potentially eligible for inclusion underwent full-text review according to the predefined inclusion and exclusion criteria. Duplicate records were removed across overlapping search strategies (Figure 1).

**Figure 1:**
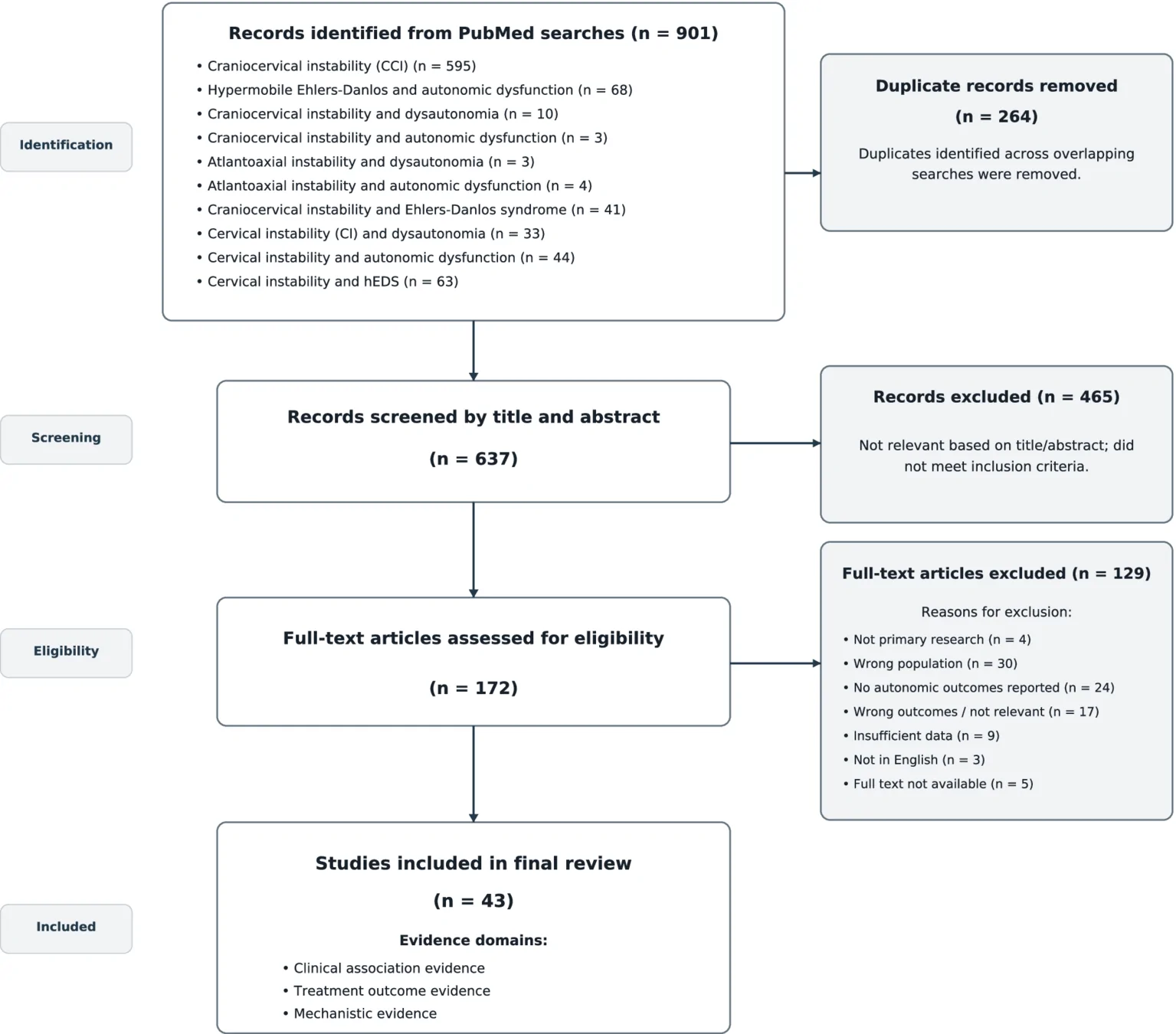
Flow diagram of literature identification, screening eligibility assessment, and study inclusion.

### Inclusion criteria

Studies were considered for inclusion if they addressed one or more of the following topics:

1. The association between CCI or AAI and symptoms of autonomic dysfunction
2. Autonomic dysfunction in patients with hEDS, or other related connective tissue disorders
3. Associations between hEDS and CCI or AAI
4. Proposed pathophysiologic mechanisms linking cervical or craniocervical pathology to autonomic dysfunction, including brainstem compression, vagal nerve involvement, neurovascular compression, or altered cerebral perfusion
5. Autonomic outcomes following surgical stabilization or other nonsurgical management of CCI/AAI
6. Diagnostic criteria or imaging findings relevant to CCI/AAI

### Exclusion criteria

Studies were excluded if they did not provide evidence relevant to the relationships among CCI/AAI, hEDS, and autonomic dysfunction or to the proposed mechanisms examined in this review. Articles were also excluded if they evaluated populations unrelated to the conditions of interest, did not report relevant autonomic or clinical outcomes, lacked sufficient data to contribute to the narrative synthesis, were not available in English, or if the full text was unavailable. Duplicate records identified across overlapping search strategies were removed prior to screening.

### Data extraction and synthesis

For each included study, the following data were extracted:

- Study design, study population, sample size, presence of CCI or AAI, presence of hEDS, presence of related connective tissue disorders, interventions, autonomic outcomes, and key findings.

The literature was subsequently organized into five primary categories. These categories included:

1. Autonomic dysfunction in hEDS
2. CCI/AAI in hEDS
3. Associations between CCI/AAI and autonomic dysfunction
4. Autonomic outcomes following surgery/stabilization of CCI/AAI
5. Proposed mechanisms linking cervical instability to autonomic dysfunction

Due to the heterogeneity of study designs, diagnostic criteria, interventions, and outcome measures, a quantitative analysis was not performed. Instead, findings were synthesized narratively to identify recurring themes, proposed mechanisms, and gaps in the current literature.

## Results

### Dysautonomia in hEDS

Multiple studies demonstrated a high prevalence of autonomic dysfunction among patients with hypermobile Ehlers-Danlos syndrome (hEDS) (Novak et al., 2025; Martinez et al., 2021; Ruiz Maya et al., 2021; Tale et al., 2026; Miller et al., 2020; Celletti et al., 2020; Peebles et al., 2022). Autonomic testing identified postural orthostatic tachycardia syndrome (POTS), orthostatic intolerance (OI), abnormal baroreflex sensitivity, and impaired cerebral blood flow in these populations (Novak et al., 2025; Celletti et al., 2020; Peebles et al., 2022). In a cohort of 102 patients with hEDS, 48% met the criteria for POTS diagnoses and 25.5% presented with OI (Celletti et al., 2020). A controlled study of 45 women also demonstrated greater orthostatic symptom burden among patients with hEDS (Peebles et al., 2022), while a study of 91 patients with POTS found that 31% met criteria for hEDS and another 24% demonstrated joint hypermobility (Miller et al., 2020). Larger observational cohorts further supported a high prevalence of autonomic symptoms and abnormalities in hEDS patients (Novak et al., 2025; Martinez et al., 2021; Ruiz Maya et al., 2021; Tale et al., 2026). Collectively, these findings support an association between hEDS and autonomic dysfunction (Novak et al., 2025; Martinez et al., 2021; Ruiz Maya et al., 2021; Tale et al., 2026; Miller et al., 2020; Celletti et al., 2020; Peebles et al., 2022) (Table 1).

**Table 1:** Clinical evidence linking hEDS/HSD, CCI/AAI, and autonomic dysfunction.

Authors	Design	*N*	Population	Evidence	Key finding
Novak et al. (2025)	Retrospective study	270	hEDS	hEDS → AD	>90% reported autonomic symptoms; reduced cerebral blood flow and mild autonomic failure were frequently identified
Collins Hutchinson et al. (2025)	Observational study	84	hEDS	hEDS → AD	Severe autonomic symptom burden; >50% had POTS and migraine
Martinez et al. (2021)	Observational study	148	hEDS	hEDS → AD	hEDS patients and patients with generalized joint hypermobility had similar autonomic symptom profiles
Tariq et al. (2025)	Observational study	385	hEDS	hEDS → AD	Orthostatic intolerance was significantly associated with cognitive complaints
Ruiz Maya et al. (2021)	Observational study	144	hEDS	hEDS → AD	Cardiovascular dysautonomia was associated with hEDS
Tale et al. (2026)	Observational study	199	hEDS	hEDS → AD	Significant cardiovascular dysautonomia was identified, with greater symptom burden in hEDS
Miller et al. (2020)	Cross-sectional study	91	POTS	AD → hEDS	31% of POTS patients met hEDS criteria; an additional 24% had generalized joint hypermobility
Celletti et al. (2020)	Observational study	102	hEDS	hEDS → AD	POTS occurred in 48%, OI in 25.5%, with abnormal autonomic regulation/baroreflex findings
Peebles et al. (2022)	Controlled study	45	hEDS, controls	hEDS → AD	hEDS patients reported greater orthostatic symptoms and underwent objective HUT/active stand testing
Bloom et al. (2026)	Retrospective study	347	EDS/CTD + CCI	hEDS → CCI	Large surgical cohort demonstrate clinically significant CCI in patients with EDS/CTDs
Spiessberger et al. (2020)	Retrospective study	26	EDS + CCI	hEDS → CCI	EDS patients with CCI underwent craniocervical fusion with successful radiographic correction
Ruhoy et al. (2025)	Retrospective study	717	Chiari/CCI population; 460 hEDS	hEDS → CCI/AD	64% had hEDS and 404 underwent surgery for CCI; hEDS was associated with greater relevant comorbidity burden
Zhao et al. (2022)	Retrospective study	12	hEDS + CM1/CCI	hEDS → CCI	hEDS patients developed CCI following Chiari decompression and subsequently underwent occipitocervical fusion
Midtlien et al. (2024)	Retrospective study	86	CTD + cerebral venous outflow disorder	CCI ↔ AD	POTS occurred in 55.8%, dysautonomia in 45.3%, and CCI in 37.2%
Henderson Sr et al. (2021)	Retrospective study	20	CTD + AAI	AAI → AD	All had lightheadedness; 15/20 had refractory syncope/presyncope
Lee et al. (2026)	Case series	8	hEDS + CCI	CCI → AD	CCI following hyperextension injury was accompanied by dizziness, brain fog, and dysautonomia

### Craniocervical instability in hEDS

CCI and atlantoaxial instability (AAI) were also reported across multiple studies in patients with hEDS and other connective tissue disorders (Mao et al., 2022; Lohkamp et al., 2022; Hauser et al., 2026; Bloom et al., 2026; Spiessberger et al., 2020; Ruhoy et al., 2025; Zhao et al., 2022; Henderson Sr et al., 2024; Henderson Sr et al., 2019; Rock et al., 2023). Surgical and observational cohorts described ligamentous laxity and clinically significant instability requiring stabilization in subsets of these patients (Bloom et al., 2026; Spiessberger et al., 2020; Ruhoy et al., 2025; Zhao et al., 2022; Henderson Sr et al., 2024; Henderson Sr et al., 2019; Rock et al., 2023). A 347-patient series evaluated surgical screening for CCI secondary to hEDS and other connective tissue disorders (Bloom et al., 2026), while additional cohorts demonstrated CCI that required fusion procedures among patients with hEDS (Spiessberger et al., 2020; Ruhoy et al., 2025; Zhao et al., 2022; Henderson Sr et al., 2024; Henderson Sr et al., 2019; Rock et al., 2023). These findings support an association between hypermobility and craniocervical instability, although diagnostic criteria and indications for surgical intervention was heterogeneous and varied between studies (Mao et al., 2022; Lohkamp et al., 2022) (Table 2).

**Table 2:** Treatment outcomes and mechanistic evidence linking cervical instability to autonomic dysfunction.

Authors	Design	*N*	Population	Intervention/Mechanism	Autonomic-relevant finding
Henderson Sr et al. (2024)	Retrospective study	53	hEDS + CCI	Craniocervical fusion	Significant improvement in syncope, orthostatic grading, nausea, dizziness, vertigo and fatigue
Henderson Sr et al. (2021)	Retrospective study	20	CTD + AAI	C1-C2 fusion	Significant improvement in lightheadedness (*p* = 0.003), presyncope (*p* = 0.006), and syncope (*p* = 0.03)
Bohra et al. (2025)	Case series	21	EDS + CCI + AD	Halo-vest traction; 16/21 subsequently underwent fusion	Significant improvement across neurologic, cardiovascular, gastrointestinal, and functional symptom domains
Henderson Sr et al. (2019)	Case series	20	hEDS + CCI + AD	Surgical stabilization	Long-term postoperative improvement in autonomic-associated symptoms
Gensemer et al. (2024)	Literature review + Case report	1	hEDS + CCI + AD	Surgical stabilization	Significant improvement in POTS/dysautonomia symptoms
Chu et al. (2021)	Case report	1	CCI + OI	Conservative/non-operative management	Reported resolution of cervicogenic dizziness, pain, and orthostatic intolerance
Rock et al. (2023)	Case series	67	EDS + CCI/AAI	Decompression/Stabilization	67.2% reported significant symptomatic improvement; autonomic-specific outcomes were not isolated
Hauser et al. (2025a)	Retrospective cohort/review	234	Cervical instability + Autonomic symptoms	Vagus nerve/IJV mechanism	Cervical structure, vagus nerve CSA and IJV patency evaluated in patients with dizziness, lightheadedness and fatigue
Hauser et al., 2024	Retrospective study	227	Cervical instability	IJV compression mechanism	Cervical structural abnormalities and IJV/optic nerve sheath findings associated with neurologic symptoms
Hauser et al. (2025b)	Observational study	203	Cervical instability + Vagus nerve/IJV mechanism	Vagus nerve/IJV mechanism	C1-C2 instability in 86.2%, IJV narrowing in 99.5%, and reduced vagus nerve CSA in 95.6%
Hauser et al. (2026)	Observational study	84	Cervical Instability + Vagus nerve/IJV mechanism	Cervical/Vagus nerve /IJV mechanism	Frequent cervical instability with reduced vagus nerve CSA and IJV narrowing at C1
Li et al. (2002)	Case series	12	Cervical Instability + Surgery	Cervical collar immobilization and surgery	7/12 had radiographic cervical instability; sympathetic symptoms improved following immobilization and surgery

### CCI/AAI and autonomic dysfunction

Evidence directly connecting CCI/AAI with autonomic dysfunction was more limited but demonstrated consistent clinical overlap (Midtlien et al., 2024; Henderson Sr et al., 2021; Lee et al., 2026; Henderson Sr et al., 2019). Among 86 patients with connective tissue disorders and cerebral venous outflow disorders, POTS was present in 55.8%, dysautonomia in 45.3%, and CCI in 37.2% (Midtlien et al., 2024). Another study of 20 patients with connective tissue disorders and AAI, all reported lightheadedness and 15 experienced refractory syncope or presyncope (Henderson Sr et al., 2021). Smaller case series and reports also described orthostatic intolerance, dizziness, syncope, and other symptoms of autonomic dysfunction in patients with cervical or craniocervical instability (Lee et al., 2026; Henderson Sr et al., 2019).

### Outcomes following stabilization

Several studies reported improvement in symptoms associated with autonomic dysfunction following stabilization of CCI/AAI (Henderson Sr et al., 2021; Henderson Sr et al., 2024; Bohra et al., 2025; Henderson Sr et al., 2019; Gensemer et al., 2024; Chu et al., 2021). Following C1-C2 fusion in a 20-patient AAI cohort, significant improvements occurred in lightheadedness (*p* = 0.003), presyncope (*p* = 0.006), and syncope (*p* = 0.03) (Henderson Sr et al., 2021). A retrospective study of 53 patients undergoing craniocervical fusion also demonstrated significant improvements in syncope and orthostatic grading scores, along with dizziness, nausea, vertigo, and fatigue (Henderson Sr et al., 2024). Additional case series reported improvement in autonomic, cardiovascular, gastrointestinal, and neurologic symptoms following halo traction or surgical stabilization (Bohra et al., 2025; Henderson Sr et al., 2019; Gensemer et al., 2024; Chu et al., 2021). However, these studies were mainly retrospective and frequently utilized patient-reported outcomes rather than objective autonomic measures (Henderson Sr et al., 2024; Bohra et al., 2025; Henderson Sr et al., 2019; Gensemer et al., 2024; Chu et al., 2021; Rock et al., 2023). Although symptomatic improvement following stabilization was found in this study, it does not establish a causal relationship between CCI/AAI and autonomic dysfunction.

### Proposed mechanisms

Several studies proposed mechanisms through which upper cervical instability could contribute to autonomic dysfunction (Hauser et al., 2026; Henderson Sr et al., 2019; Hauser et al., 2025a; Hauser et al., 2024; Hauser et al., 2025b; Li et al., 2002). These predominantly involved repeated distortions of craniocervical anatomy leading to vagus nerve dysfunction, internal jugular vein compression, altered cerebral venous outflow, or effects on cervicomedullary structures (Hauser et al., 2026; Henderson Sr et al., 2019; Hauser et al., 2025a; Hauser et al., 2024; Hauser et al., 2025b). Studies using cervical imaging and ultrasound reported associations between cervical instability, reduced vagus nerve cross-sectional area, and internal jugular vein narrowing, while showing similar findings in hEDS populations (Hauser et al., 2026; Hauser et al., 2025a; Hauser et al., 2024; Hauser et al., 2025b). These findings provide biological and mechanistic plausibility for a relationship between cervical instability and autonomic dysfunction; however, much of the available evidence remains observational or hypothesis-generating (Hauser et al., 2026; Hauser et al., 2025a; Hauser et al., 2024; Hauser et al., 2025b; Li et al., 2002). The mechanisms proposed in this review should be considered as biologically plausible hypotheses rather than established causal pathways.

## Discussion

### Principal findings

Analysis of the literature shows that there is likely an association between hEDS and autonomic dysfunction (Novak et al., 2025; Ruiz Maya et al., 2021; Miller et al., 2020; Celletti et al., 2020; Peebles et al., 2022), as well as an association between hEDS and CCI/AAI (Mao et al., 2022; Lohkamp et al., 2022; Bloom et al., 2026; Spiessberger et al., 2020; Ruhoy et al., 2025; Zhao et al., 2022; Henderson Sr et al., 2024; Henderson Sr et al., 2019). Evidence directly linking CCI/AAI with autonomic dysfunction is much more limited (Midtlien et al., 2024; Henderson Sr et al., 2021; Lee et al., 2026; Henderson Sr et al., 2019), as substantial heterogeneity exists in study design, diagnostic criteria, and autonomic outcome measurements. Nevertheless, these associations have not been extensively reported in the past, warranting future investigation.

Several studies also reported improvement in autonomic-associated symptoms following craniocervical or atlantoaxial surgical stabilization (Henderson Sr et al., 2021; Henderson Sr et al., 2024; Bohra et al., 2025; Henderson Sr et al., 2019; Gensemer et al., 2024; Chu et al., 2021). Though these results were largely heterogeneous both in their study design and in the type of surgical procedures reported, they still provide preliminary evidence supporting the mechanism of CCI/AAI and cervical instability (CI) being large contributors to autonomic dysfunction through the compression of neurovascular structures. Importantly, though the available evidence is promising, it does not establish a causal relationship.

The most common symptoms of dysautonomia are orthostatic symptoms, presyncope/syncope, palpitations, tachycardia, blood pressure instability, fatigue, “brain fog,” headaches, GI dysmotility, and thermoregulatory dysfunction (Peltier, 2024). For purposes of this review, these symptoms were considered when studies did not explicitly diagnose “autonomic dysfunction.” Given variability in autonomic symptom reporting and objective testing across studies, these findings should be interpreted cautiously.

### Potential mechanisms linking CCI/AAI to dysautonomia

Several potential mechanisms may explain the association between CCI/AAI and autonomic dysfunction. Patients with hEDS seem to be at increased risk of developing CCI/AAI (Mao et al., 2022; Lohkamp et al., 2022; Bloom et al., 2026; Spiessberger et al., 2020; Ruhoy et al., 2025; Zhao et al., 2022). In patients with cervical instability, excessive motion at the craniocervical junction may contribute to compression or irritation of adjacent neurovascular structures (Henderson Sr et al., 2019). Proposed mechanisms identified in this review include cervicomedullary compression, vagus nerve compression, internal jugular vein compression with altered cerebral venous outflow, and impaired cerebral perfusion (Hauser et al., 2026; Midtlien et al., 2024; Henderson Sr et al., 2019; Hauser et al., 2025a; Hauser et al., 2024; Hauser et al., 2025b). These mechanisms provide biological plausibility for autonomic symptoms in affected patients, especially with the increased reported occurrence of both cervical instability and autonomic dysfunction that was found in patients with hEDS. However, current mechanistic evidence remains observational and hypothesis-generating (Hauser et al., 2026; Hauser et al., 2025a; Hauser et al., 2024; Hauser et al., 2025b). A direct causal pathway between cervical instability and autonomic dysfunction is being presented but not yet established.

### Clinical implications

CCI/AAI may represent a structural contributor to autonomic dysfunction in a subset of hEDS patients, particularly those with symptoms of autonomic dysfunction and significant cervical and neurologic manifestations (Midtlien et al., 2024; Henderson Sr et al., 2021; Henderson Sr et al., 2019; Hauser et al., 2025a). Recognition of this potential association may lead to more comprehensive evaluation of structural pathology, leading patients to quicker diagnoses, and prompt treatment.

Several studies also reported improvement in autonomic and neurologic symptoms following surgical stabilization (Henderson Sr et al., 2021; Henderson Sr et al., 2024; Bohra et al., 2025; Henderson Sr et al., 2019; Gensemer et al., 2024; Chu et al., 2021). Other studies reported that patients also showed improvement following conservative management strategies like activity modification, symptom-directed medical management, physical and manual therapy (Garreth Brittain et al., 2024). Surgical stabilization should be reserved for patients who fail conservative therapy and for those with severe and/or progressive neurological symptoms and weakness combined with radiographic evidence of instability. Since craniocervical fusion is an invasive and irreversible intervention with the potential for postoperative complications, careful patient selection and individualized risk–benefit assessment are essential (Mao et al., 2022; Lohkamp et al., 2022; Henderson Sr et al., 2024).

However, these findings should not be interpreted as evidence supporting any specific treatment approach over another. Rather, they highlight the clinical importance of identifying cervical instability when present, and the need for more robust investigations into determining which patients benefit from each specific management strategy.

### Diagnostic challenges

A major challenge identified in this analysis was a lack of standardized diagnostic criteria for CCI/AAI, and autonomic dysfunction. Diagnostic criteria for CCI/AAI, varied across studies, with differences in the imaging modalities, radiographic measurements, and thresholds used to define instability (Mao et al., 2022; Lohkamp et al., 2022). Furthermore, discrepancy in the literature exists over the best imaging modality for diagnosis of CCI/AAI. Some experts argue that the best diagnostic modalities for evaluation of cervical instability are upright MRIs or flexion/extension CT scans (Jovin et al., 2020). This variability limits comparison across studies in this review and remains an important barrier to establishing the clinical significance of CCI/AAI.

Clinical evaluation of CCI/AAI requires a combination of symptoms, neurologic findings, and radiographic evidence of stability. Patients with CCI/AAI often present with severe headache, neck pain and objective neurologic deficits (Mao et al., 2022; Lohkamp et al., 2022). Imaging may include dynamic radiographs, CT and MRI, with commonly reported measurements including the clivo-axial angle, Harris measurement, Grabb-Mapstone-Oakes measurements, and the angular displacement of C1-C2 (Mao et al., 2022; Lohkamp et al., 2022). These measurements assess for abnormal motion at the craniocervical junction and upper cervical spine that may lead to neurologic compromise. Importantly, radiographic findings should be interpreted along with clinical symptoms and neurologic findings for accurate diagnoses (Mao et al., 2022; Lohkamp et al., 2022).

Autonomic dysfunction is a heterogeneous diagnosis and encompasses a broader spectrum of disorders than POTS alone. Evaluation for autonomic dysfunction may include head-up tilt testing, heart rate and blood pressure responses, cardiovagal testing, and other measures of sympathetic and parasympathetic function (Peltier, 2024; Celletti et al., 2020; Peebles et al., 2022). However, the studies included in this review varied considerably in how autonomic dysfunction was defined and assessed.

Diagnostic heterogeneity also exists across hypermobility disorders (DePace et al., 2025; Hakim, 2004). The Beighton score is commonly used to assess generalized joint hypermobility, but it is not diagnostic for hEDS by itself (DePace et al., 2025). Diagnosis of hEDS is currently clinical and requires additional criteria beyond generalized joint hypermobility (DePace et al., 2025; Forghani et al., 2025;Jovin et al., 2020). Genetic testing can be done for some forms of Ehlers-Danlos syndrome but is not routinely performed (Atwell et al., 2021). Hypermobility spectrum disorders often encompass patients with symptomatic hypermobility that do not meet criteria for hEDS (DePace et al., 2025; Atwell et al., 2021; Wang and Serrano-Ardila, 2026).

Overall, greater standardization for CCI/AAI definitions, imaging, diagnostics and clinical evaluation protocols for autonomic assessments must be established to further understand these relationships.

## Limitations

Several limitations of the current evidence restrict our ability to make substantial conclusions from this review. Most of the studies used were retrospective or small case series. This allows the risk of selection and reporting bias to influence results, particularly among surgical and posttreatment cohorts. Additionally, objective pre- and post-intervention autonomic testing was limited, which led studies to rely on patient-reported symptoms associated with dysautonomia.

Autonomic dysfunction was inconsistently defined across studies. Therefore, common reported symptoms of dysautonomia were used as proxies when objective autonomic diagnoses were unavailable. Similarly, heterogenous definitions for CCI/AAI limit the ability to consistently characterize cervical instability across the study populations. Mechanistic evidence was also mostly observational and hypothesis-generating, limiting conclusions regarding causality.

Lastly, this review was narrative by design, limited to English-language studies there were identified through PubMed, and did not include a formal risk-of-bias assessment. These methodological limitations may have resulted in relevant evidence being excluded and should be kept in mind when interpreting findings.

## Future directions

Future studies in these populations should focus on prospective cohort studies utilizing standardized definitions of CCI/AAI and autonomic dysfunction. Patients with hSD and hEDS should be compared to patients with and without CCI/AAI, while obtaining objective autonomic testing before and after stabilization procedures are performed. There should also be studies analyzing the severity of radiographic instability with autonomic abnormalities to further investigate these relationships, while also directly investigating levels of vagus nerve, internal jugular vein (IJV) and cerebral perfusion, to see if these can directly be the causes of autonomic dysfunction in these patients.

## Conclusion

The current evidence supports and justifies further investigation of CCI/AAI as a potential contributor to dysautonomia but is insufficient to conclude that this is a causal relationship. Given the observed associations between hEDS, CCI/AAI, and autonomic dysfunction, prospective studies using standardized diagnostic criteria and objective autonomic measures are required to better understand these relationships.
